# Tumor Targeting via Integrin Ligands

**DOI:** 10.3389/fonc.2013.00222

**Published:** 2013-08-30

**Authors:** Udaya Kiran Marelli, Florian Rechenmacher, Tariq Rashad Ali Sobahi, Carlos Mas-Moruno, Horst Kessler

**Affiliations:** ^1^Institute for Advanced Study (IAS) and Center for Integrated Protein Science (CIPSM), Department Chemie, Technische Universität München, Garching, Germany; ^2^Department of Chemistry, Faculty of Science, King Abdulaziz University, Jeddah, Saudi Arabia; ^3^Biomaterials, Biomechanics and Tissue Engineering Group, Department of Materials Science and Metallurgical Engineering, Technical University of Catalonia (UPC), Barcelona, Spain

**Keywords:** integrins, RGD, tumor, targeted delivery, αvβ3, αvβ5, α5β1 and αvβ6

## Abstract

Selective and targeted delivery of drugs to tumors is a major challenge for an effective cancer therapy and also to overcome the side-effects associated with current treatments. Overexpression of various receptors on tumor cells is a characteristic structural and biochemical aspect of tumors and distinguishes them from physiologically normal cells. This abnormal feature is therefore suitable for selectively directing anticancer molecules to tumors by using ligands that can preferentially recognize such receptors. Several subtypes of integrin receptors that are crucial for cell adhesion, cell signaling, cell viability, and motility have been shown to have an upregulated expression on cancer cells. Thus, ligands that recognize specific integrin subtypes represent excellent candidates to be conjugated to drugs or drug carrier systems and be targeted to tumors. In this regard, integrins recognizing the RGD cell adhesive sequence have been extensively targeted for tumor-specific drug delivery. Here we review key recent examples on the presentation of RGD-based integrin ligands by means of distinct drug-delivery systems, and discuss the prospects of such therapies to specifically target tumor cells.

## Introduction

Cancer diagnosis, therapy, and monitoring represent fundamental topics of research in medicine and are of utmost importance in healthcare of today’s society. An efficient cancer therapy should possess exceptional abilities not only to ensure a complete removal of the tumor but also to prevent its spreading and invasion to other tissues by metastasis. Current clinical approaches to treat cancer include, and often combine, surgery, chemotherapy, radiation therapy as well as immunotherapy. However, these methods in general still fail to treat highly aggressive metastatic cancers, and present some serious limitations. For instance, irradiation of tumors may damage adjacent healthy tissues, and chemotherapy, which is based on a non-specific systemic distribution regime, requires high drug dosage and promotes severe adverse side effects. For example, the administration of Paclitaxel (PTX), a drug used for the treatment of lung, ovarian, and breast cancers, has been associated with unwanted effects such as hypersensitivity reactions, myelosuppression, and neurotoxicity (1, 2), among others. Doxorubicin (DOX), another drug used in cancer chemotherapy, has also been described to have cardiotoxic side effects (3, 4). Moreover, chemotherapy might turn inefficient due to acquired chemoresistance as exemplified in the case of Gemcitabine – prime therapeutic used to treat pancreatic cancers (5), for DOX (3) and also for PTX (6, 7).

**Figure 1:**
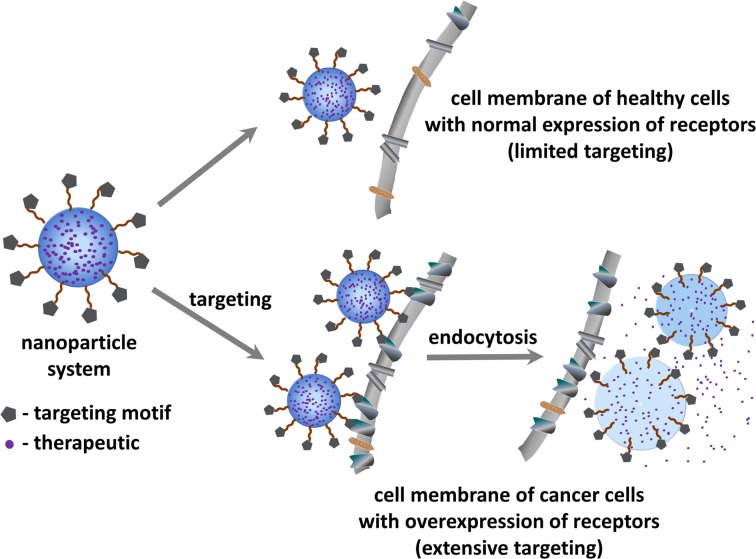
**Schematic representation of the principle of tumor targeted drug delivery for treating cancer**.

Tumor targeted drug-delivery (Figure 1) represents a promising approach to overcome some of the above mentioned limitations (8). This strategy aims to specifically guide and direct anticancer therapeutics (or imaging agents) to tumor cells without interfering with normal tissues. Such targeted approach relies on the fact that tumor vasculature and tumor cells display a well-differentiated pattern of (over-)expression of specific receptors (i.e., receptors required for tumor angiogenesis), which is consistent with the concept of “Vascular Zip Codes” (9, 10). Targeted drug-delivery methods hence employ small molecules or monoclonal antibodies selective to receptors that are proven to be abnormally expressed on tumors. The conjugation of anticancer drugs to these selective ligands will allow a preferential or selective delivery of the drug to the tumor.

As a result, this technique benefits from several advantages: (i) non-specific interactions with normal tissues are reduced, and thus the adverse side-effects associated to conventional chemotherapy can be minimized. (ii) Site-directed drug release leads to higher local concentrations at the diseased tissue and thus allows dosage reduction. (iii) Acquired chemoresistance can potentially be reduced by co-delivering other therapeutics capable of regulating cancer multi-drug resistance (MDR). To avail these advantages, well accessible cell surface receptors are preferred over intracellular targets where (complex) drug internalization mechanisms need to be taken into consideration. In this regard, one of the most intensely referred class of proteins for targeted therapy is the integrin family (11).

**Figure 2:**
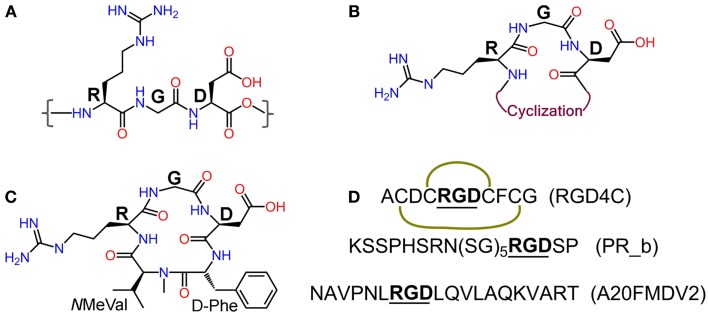
**(A)** Integrin recognition motif RGD; **(B)** schematic representation of cyclic RGD (*c*RGD); **(C)** Cilengitide – *c*(RGDf-*N*MeVal); **(D)** peptide sequences of RGD4C (the green curves indicate disulfide bridges), α5β1 ligand PR_b, and αvβ6 ligand A20FMDV2.

Integrins are heterodimeric transmembrane glycoproteins consisting of an α and a β subunit. In total, 24 different subtypes of integrins that are constituted from 18 α and 8 β subunits have been discovered to date (12). Almost half of them bind to various extra cellular matrix (ECM) proteins such as fibronectin, vitronectin, and collagen through the tripeptide motif Arg-Gly-Asp = RGD [(13), Figure 2], and are vital in the adhesion, signaling, migration, and survival of most cells (14). Integrins have also very important roles in cancer progression and some subtypes have been described to be highly over-expressed on many cancer cells. This is the case of integrins αvβ3, αvβ5, and α5β1, which are crucial mediators of angiogenesis in cancer (8, 15–17). Underlying cause for this is the elevated demand by the enlarging tumor for adequate supply of necessary nutrients and oxygen. In order to meet these demands through blood supply, tumor tissue with a rapidly overgrowing number of cells, signals [via growth factors like vascular endothelial growth factor (VEGF) or basic fibroblast growth factor (bFGF)] for increased angiogenesis, a state known as “angiogenic switch.” Sprouting of new blood vessels and overexpression of integrins in tumor tissues and vasculature are thus key features in the pathophysiology of cancer. Other integrins such as αvβ6 and α6β4 are also observed to be expressed on tumor cells (8). Another pivotal function of integrins is the promotion of cell migration by virtue of their binding to ECM components. This phenomenon is responsible for the process of tumor proliferation, migration, invasion, and metastasis (18). These functional aspects together with the high expression levels found on tumor cells have converted integrins into very interesting proteins for targeted cancer diagnosis and therapy studies.

Our review shortly recapitulates recent developments in integrin targeted cancer therapy, with special focus on targeted delivery of chemotherapy or gene therapy via non-viral vectors like nanoparticles (NPs), micelles, vesicles, or other systems grafted with RGD-based integrin ligands. Considering the vastness of the topic, we have only cited a limited amount of recent works. For previous studies and developments in this field other detailed reviews are available (19–22). Applications based on integrin targeting antibodies and therapies involving the blocking of integrin functions with antagonists and other ligands are not subject of this review.

### Integrin ligands and integrin targeting

Since the discovery of the integrin recognizing RGD motif by Ruoslahti et al. (13, 23), extensive research has been carried out to develop RGD-based peptide and peptidomimetic integrin ligands (24). Various synthetic strategies have been applied to develop RGD peptide analogs with enhanced biological properties and pharmacokinetics like affinity and selectivity for different integrin subtypes, metabolic stability, and biodistribution. These strategies include the introduction of amino acids flanking the tripeptidic RGD sequence, cyclization, and variation of stereochemical configuration of the constituent amino acids (25), and N-methylation (26, 27) (Figure 2). Cilengitide – *c*(RGDf-*N*MeVal) (Figure 2), a very potent antagonist of αvβ3, was developed by using some of these approaches and has been clinically tested by Merck primarily for treatment of glioblastoma multiforme (28, 29). Despite promising preliminary data, its use as anticancer therapeutic has been discontinued due to failure in phase-III clinical trials (Merck press release on Cilengitide studies: http://www.merck.de/de/presse/extNewsDetail.html?newsId=C47977D13865FCB9C1257B1D001EF9CA&newsType=1). Other well-known RGD peptides are *c*RGDfV (25) – the parent peptide for Cilengitide, *c*RGDfK (30), and RGD4C (ACDCRGDCFCG) (31). RGD4C is susceptible to be expressed by recombinant methods into proteins and viruses for their targeted delivery. Targeting integrins using *c*RGDfX, *c*RGDeV, *c*RGDyV, and other peptides or peptidomimetics (Figure 2) has also been reported in the literature.

### Targeted drug delivery

Targeted delivery can be accomplished by two approaches: the direct conjugation of the targeting motif to the drug or the use of drug vehicular systems grafted with the targeting motif. Of these, the use of carrier systems offers several advantages compared to direct conjugation methods:

1. Carrier systems have the capacity to present multiple ligands on each particle. This facilitates effective targeting via multiple and simultaneous interactions between the ligands and the receptors, exploiting the concept of multivalency.
2. Vehicular systems may keep the drug unexposed to physiological systems, thereby protecting it from degradation or alteration, and more importantly, minimizing undesirable non-specific interactions of the drug with normal tissues. Therefore, these systems may remarkably reduce the side effects of the drug.
3. Targeted carrier systems usually are internalized via receptor-mediated endocytosis and the drug is directly released within cell. This is more effective to attain higher in-cell drug concentrations for amplified therapeutic activity.
4. Being larger in size (∼>100 nm) than classical drugs, carrier systems are not filtered off by renal pathways (size limit for renal filtration ∼5 nm). This enables a prolonged half-life time of carrier particles in the blood stream and allows for a gradual release of the drug over longer periods of time. Such release kinetics avoid high systemic concentrations of the drug and improves the effectiveness of the administered dose.
5. The abnormal architecture and permeability of tumor vasculature promotes extravasation of the particles that are in blood circulation. This phenomenon is called enhanced permeability and retention (EPR) effect. Facilitated by this passive transport mechanism, the nano-sized vehicular systems enter into tumor tissues. However, the quick clearance of these NPs from the tissue is prevented by their large size and lead to prolonged retention times in tumor. Hence, the double targeting – passive and active receptor-mediated targeting, enhances therapeutic efficacy.

Among the carrier systems, viral vectors such as retroviruses and adenoviruses have been successfully developed and found to be efficient in targeted gene therapy (32). However, their use is associated with several disadvantages that have precluded their clinical application. In the first place, they can produce unwanted immune responses (33). Also, it is not easy to express viruses composed with targeting moieties that contain unnatural amino acids or chemically modified scaffolds. Moreover, viral vectors can only be used for gene therapy and are not suitable for delivery of chemotherapeutics. Last but not least, they also carry a negative public perception concerning safety (33, 34). Therefore, development of non-viral targeting vectors is a preferred alternative in targeted therapy. In this regard, various kinds of polymer-based nanocarriers have been developed for tumor targeting using integrin ligands including the use of RGD coated virus like particles (VLPs) which use only the capsid of the viruses (35). In the following sections, some representative examples are discussed according to the targeted integrin subtype.

## Targeting αvβ3 and αvβ5 Integrins

As previously introduced, the αvβ3 integrin subtype plays a major role in angiogenesis, tumor neovascularization, and tumor metastasis (8). The angiogenic pathways dependent on αvβ3 have been described to be induced by bFGF or tumor necrosis factor α (TNF-α). Its expression is upregulated on angiogenic endothelial cells (36–38) and on various tumor cell lines (39, 40). Antagonistic inhibition of αvβ3 integrin has been shown to suppress angiogenesis (41) and to induce apoptosis (42). The well-established biological roles, high expression on tumor tissues, and the availability of ligands with high affinity, have set αvβ3 the most extensively studied integrin for tumor targeting. The integrin αvβ5 is also involved in angiogenesis but through a distinct pathway stimulated by VEGF or transforming growth factor α (TGF-α) (16). Since most RGD-containing peptidic αvβ3 antagonists also recognize αvβ5, although usually with a lower affinity, these two integrin subtypes are discussed together.

### Targeted delivery of chemotherapy using polymeric vehicles

Encapsulation of drugs in polymer-based carrier systems is a practical approach to protect them from degradation in biological system. Furthermore, these systems may reduce the systemic toxicity of the drug and also enhance their safe elimination from the physiological system. In addition, these vehicles often ameliorate the drug’s pharmacokinetic profile and biological distribution within the organism. Phospholipid or polypeptide-based polymers are commonly employed to prepare drug-delivery vehicles as they are akin to biological molecular components and thus display low toxicity and are easily biodegradable. Since the physicochemical properties of these polymers can be easily tuned to produce liposomes, micelles, or NPs, via well-established protocols, these materials are frequently used to construct drug-delivery vehicles. In fact, liposomes have already been used for the formulation and delivery of DOX (4). These vehicles may additionally be PEGylated to improve their aqueous solubility and to reduce non-specific interactions with plasma proteins and membranes. Besides encapsulation, drugs can as well be bound to these systems by chemical methods. This enables drug stability and also secured pH-sensitive release of drugs *in situ*. These sorts of carrier systems have been equipped with integrin targeting ligands and experimented for their capabilities as targeted drug-delivery systems in cancer treatment. Some illustrative recent works are listed in Table 1.

**Table 1:** **Outline of representative recent examples of polymer-based targeted delivery studies using αvβ3 and/or αvβ5 integrin ligands**.

Carrier system	Targeting motif	Drug	Cellular system	Results and characteristics (reference)
Cholesterol/DOPE/DSPC/DSPE-(PEO)4-cRGDfK/DSPE-mPEG2000	*c*RGDfK	DOX	R40P murine pancreatic and SN12C renal carcinoma cells	Fifteen fold increase in drug efficacy relative to animals treated with free drug (95)
PLG-PEG micelles	*c*RGDfC	DOX	U87MG human glioblastoma cells	pH-sensitive drug release, higher cellular uptake, higher accumulation at tumor sites as monitored by positron emission tomography (PET) and *ex vivo* fluorescence experiments (96)
PLGA-4-arm-PEG branched NPs	*c*RGDfC	–	Pancreatic tumor in mice and U87MG glioma cells	Efficient uptake by U87MG glioma cells over-expressing αvβ3. Highest accumulation at tumor site as monitored by whole body imaging. Low *in vivo* inherent physiological toxicity for the NPs (97)
PGA-PTX-E-[*c*(RGDfK)]2 conjugate NPs	*c*RGDfK	PTX	4T1 murine breast cancer tumors	Augmented antitumor activity and reduced systemic toxicity for PTX, blockade of endothelial cell migration to VEGF and adhesion to fibrinogen. Lysosomal enzyme assisted release of PTX is observed (98)
PLGA-PEG NPs	GRGDS and RGD peptidomimetic	PTX and DOX	HUVECs and syngenic TLT cells	High cellular uptake *in vitro*, improved anticancer efficacy and higher survival rate of mice (99)
*c*RGDyK-PEG-PLA-PTX micelle	*c*RGDyK	PTX	Intracranial glioblastoma model	2.5-Fold increase in antiglioblastoma cell cytotoxicity effect over non-targeted system, improved drug accumulation, increase in life time of diseased mice (100)
**FOR OTHER STUDIES USING PLGA-PLL NPs PLEASE SEE REF**. (101, 102)				
HPMA copolymers	*c*RGDfK	Geldanamycin	PC-3 and DU145 prostate cancer cell lines	Tumor growth inhibition activity as efficient as free drug, decrease in IC_50_ values for targeted conjugates. Improvements in biodistribution profile, both *in vitro* and *in vivo* antiangiogenic, and antitumor activities for targeted systems (103–105)
HPMA copolymers	*c*RGDfK	Docetaxel	PC-3 and DU145 prostate cancer cell lines	Inhibition of PC3, DU145 cell growth and also of HUVECs *in vitro*. *In vivo* tumor regression is also observed (106)
PCL-PEEP and Mal-PEG-PCL micelles	Tf and *c*RGDfK	PTX	BMEC and U87MG glioma cells	Double targeting by Tf and RGD ligand. Uptake of micelles increased 2.4 times for BMEC compared to micelles lacking Tf. High drug accumulation in brain upon IV injection (107)
HPAE-co-PLA/DPPE polymer NPs	Tf and *c*RGDfK	PTX	HUVECs and HeLa cells	*In vitro* cytotoxicity for NPs coated with *c*RGD is increased 10 times in αvβ3-expressing HUVECs while Tf targeting to Tf receptor over-expressed HeLa cells lead to twofold increase. pH-sensitive intracellular drug release (108)
PFC (perfluorocarbon) NPs	Non-peptidic αvβ3 antagonist	Fumagillin	Vx-2 adenocarcinoma tumor	Diminished development of tumor neovasculature and reduced tumor growth are observed at much lower drug concentrations compared to the previous concentration used in rodent and human clinical trials (109)
P(PEGMEMA) based micelles	RGD	Albendazole	OVCAR-3 ovarian cancer cells	Improved cellular uptake of polymeric micelles and 80% cell deaths at a micelle concentration of 10 μg mL^−1^ (110)

### Targeted delivery of chemotherapy using protein-based NPs

Although polymer-based vehicle systems are a common choice for drug delivery, their long-term biological toxicity might be an issue and needs to be carefully assessed. For this reason, protein-based NPs are considered an attractive alternative for targeted therapy due to their high biocompatibility, biodegradable properties, and water solubility. With regard to this, albumin is one of the proteins that has been most majorly explored for drug delivery. For example, linking *c*(RGDyK)C to albumin NPs loaded with Gemcitabine showed an increased *in vitro* and *in vivo* antitumor efficacy in BxPC-3 pancreatic cancer cell lines compared to NPs without the targeting sequence (43). The conjugation of cyclic RGD to albumin not only lead to successful targeting but also increased the intracellular uptake of NPs and Gemcitabine as monitored by florescence studies. The αvβ3-mediated uptake of the RGD-conjugated components into pancreatic cells was further confirmed by competitive inhibition studies using soluble RGD ligands. In another study (44), Fluorouracil-bearing *c*RGDfK-albumin nanospheres have shown significant improvement in binding to αvβ3-expressing HUVEC cells *in vitro*. A considerable improvement in prevention of lung metastasis and angiogenesis, and in tumor regression was observed *in vivo* in B16F10 tumor-bearing mice as compared with the activity of the free drug. The binding of nanospheres conjugated with RGD to endothelial cells was eightfold higher than that of nanospheres without RGD or conjugated with the RAD sequence (which does not bind to integrins). Similarly, enhanced homing to tumors and endothelial cell binding were reported for *c*RGDfK-PEG-albumin NPs that were linked to the antimitotic agent monomethyl-auristatin-E (MMAE) (45). These studies were carried out on HUVECs and C26 carcinoma-bearing mice. Two kinds of target systems were prepared with an RGD peptide linked to albumin either by a PEG chain (RGD-PEG-MMAE-HSA) or a short alkyl chain (RGD-MMAE-HSA). After IV administration in mice, fluorescent studies showed colocalization of both carrier systems with the tumor vasculature and tumor cells.

Besides the use of albumin as drug-delivery system, spider silk is a protein that holds great promise for application in targeted therapies. Due to its water solubility, excellent biocompatibility, and unique mechanical properties, spider silk has attracted growing interest in a number of biomedical areas. Spider silks are currently under investigation for the encapsulation and controlled release of drugs and growth factors, with so far optimistic outcomes (46). Scheibel’s group has prepared spider silks containing the integrin recognition motifs GRGDSP or *c*RGDfK by either recombinant expression or chemical methods, respectively (47). These RGD functionalized proteins have been used to generate spider silk films that retain the biophysical properties observed for silks prepared using the native proteins. Significant improvements in the attachment and proliferation of BALB/3T3 mouse fibroblasts were observed on films containing the RGD sequence but not on unmodified or RGE-containing silk. These results encourage further exploration of spider silk protein as a prospective carrier system for targeted drug delivery in cancer.

### Targeted delivery of chemotherapy using metallic NPs

Gold and other metallic NPs can be used for the polyvalent display of targeting scaffolds (48). Ease of preparation and functionalization as well as unique physicochemical properties make gold NPs very attractive systems for use in cancer diagnosis and therapy. For instance, PEGylated gold NPs coupled to a *c*RGD peptidomimetic via thiol chemistry showed good affinity and binding to αvβ3-positive PC-3 prostate cancer cells *in vitro* (49). In another study, Yang et al. have examined the utility of multifunctional PEGylated superparamagnetic iron oxide (SPIO) NPs in targeted drug delivery and PET/Magnetic Resonance Imaging (MRI) (50). To this end, *c*RGDfC and a common ^64^Cu chelator were bound to the distal ends of the PEG chains, whereas the drug, DOX, was conjugated to the SPIO particles via pH-sensitive hydrazone bonds. The *c*RGD-conjugated SPIO nanocarriers exhibited higher cellular uptake and cytotoxicity in U87MG cells compared to *c*RGD-free systems. Also, *in vivo* PET imaging of U87MG tumor-bearing mice revealed increased tumor accumulation of *c*RGD-SPIO NPs compared to *c*RGD-free counterparts. Intracellular specific drug release by SPIOs was facilitated by pH-selective cleavage of the SPIO-DOX hydrazone linkage. Such multifunctional systems that are able to simultaneously target a cell or tissue, deliver a drug, and provide a diagnosis are known as theranostics, which constitute an upcoming area of research.

### Targeted delivery of gene therapy

Delivery of gene therapy using targeted non-viral vehicles has been widely studied (20). A directed delivery of DNA or RNA fragments is required to prevent from using high doses, which otherwise can lead to off-target gene silencing effects. Using carrier systems for gene therapy is advantageous as it reduces the problems of biodegradability, nucleosomal cleavage, and size and charge limited membrane impermeability associated with the delivery of nucleic acids. As mentioned earlier, non-viral vectors are also helpful to overcome complications and safety issues described for viral vectors. Here, we briefly tabulate some recent targeted gene therapy studies (Table 2).

**Table 2:** **Outline of recent targeted gene delivery studies using αvβ3 and/or αvβ5 integrin ligands**.

Carrier system	Targeting motif	Gene	Cellular system	Results and Characteristics (reference)
PEG-PLys polyplex micelle	*c*RGDfK	Luc-pDNA	HeLa cells and 293T cells	Enhanced transfection efficiency (TE) and perinuclear accumulation of pDNA within 3 h of incubation (111)
PEG-PLys polyplex micelle: cross-linked by thiolation	*c*RGDfK	Luc-pDNA	HeLa cells and 293T cells	Improvements in TE, selection of endocytotic pathways and regulation of intracellular trafficking by *c*RGD. Preferential caveolae mediated endocytosis is observed. Thiol cross-linking helped polyplex stabilization and pDNA protection (112)
PEG-PLys polyplex micelle: cross-linked by thiolation	*c*RGDfK	sFlt-1	BxPC-3 pancreatic adenocarcinoma tumors	Upon IV injection, significant tumor-specific TE and gene expression is observed which lead to a decrease in tumor vasculature. Thiol cross-linking has to be optimized to improve results (113, 114)
PEG-PEI polyplex micelles	B6 peptide and RGD bicyclo peptide	pCMVLuc	DU145 and PC3 prostate cancer cells	Significant improvement in TE via targeting. RGD helped in initial association of polyplexes to cells whereas the internalization is observed to be mediated by TfR endocytosis (115)
PEG-PEI polyplex micelles	Non-cyclic RGD-peptidomimetic		MeWo and A549 cells	Increased binding, uptake, and luciferase transgene expression in model cells (116)
PEG-PEI polyplex micelles	*c*RGDyK	pORF-hTRAIL	Intracranial U87 glioblastoma tumor xenografts	Higher gene transfection and increased therapeutic efficiency of TRAIL are observed and is reflected in improved longevities of mice (117)
DNA/PEI-Au-RGD nanoclusters	Cap-RGD	pEGFP-Luc	HeLa cells	A 5.4- to 35-fold increase in TE corresponding to a low or high density of αvβ3 on HeLa cells. Observed TEs are far higher than that for targeted or untargeted commercial transfection vector – JetPEI. Higher concentration of gold NPs is found to be toxic (118)
PEG–oligo(ethane amino) amide polymers	B6 peptide or *c*RGDfK	pEGFP-Luc	Mouse N2A neuroblastoma and DU145 human prostate adenocarcinoma cells	Selective binding and transfection efficiency are observed which are mediated by the targeting ligands. The carrier systems however required use of endosomolytic agents for release of polyplexes from endosomes (119)
DCP-TEPA polycation liposomes	*c*RGDfK	siLuc2	B16F10-luc2 murine melanoma cells	Successful targeting, transfection, and knockdown of luc2 expression *in vitro* in B16F10-luc2 cells and also *in vivo* as monitored by imaging in mice with tumor-bearing lungs, is observed (120)
PEO-*b*-PCL micelles	RGD4C	mdr1 siRNA and DOX	MDA435/LCC6 cells resistant to DOX	The system is decorated with cell penetrating peptide (TAT) as well. Dual functional micelles showed improved cellular uptake and mdr1 activity leading to lowered P-gp expression both at the mRNA and protein levels. These effects caused reversal of MDR for DOX, which increased DOX accumulation in cytoplasm and nucleus, and enhanced DOX cytotoxicity (121)

### Phototherapy using targeted systems

Gormley et al. have tested the use of targeted gold nanorods (GNRs) for plasmonic photothermal therapy (PPTT) aiming at reducing the amount of heat required in thermal therapy (51). To this end, PEGylated GNRs were prepared and functionalized with *c*RGDfK via thiol chemistry. Studies on HUVEC and DU145 prostate cancer cells showed effective *in vitro* selective targeting of RGD-GNRs to both these cell types but not *in vivo* in a DU145 mice model. The absence of *in vivo* effects was attributed to faster clearance of GNRs from physiological system due to the presence of negative charges in *c*RGDfK-functionalized GNRs. On similar lines, for PPTT, Akhavan et al. have projected reduced single layer graphene oxide nanorods (GONRs) functionalized by amphiphilic PEG polymers containing RGD-based peptides (52). RGD-presenting GONRs showed increased radiation absorption compared to non-functionalized GONRs and also improved destruction of U87MG human glioblastoma cells at reduced doses as low as 1 μg mL^−1^. Irradiation for 8 min with near-infrared radiation at this concentration resulted in remarkable values of cell destruction (≥97%). On the contrary,<11% of cell destruction and 7% of DNA fragmentation were observed for non-targeted nanorods using the same concentration.

## Targeting the α5β1 Integrin

In addition to αvβ3 and αvβ5, an upregulated expression of α5β1 in tumor vasculature and other cancer cells has also been described (36, 53–57). α5β1 primarily recognizes fibronectin through the RGD binding motif. Kim et al. have reported that α5β1 inhibition induces cell apoptosis in endothelial cells (58) and also showed that this integrin mediates the migration of endothelial cells. Noteworthy, it has been shown that α5 might substitute the activity of αv during vasculature remodeling (59). For these reasons, targeting of this integrin has also been approached in cancer therapy.

Kokkoli and co-workers have explored α5β1 integrin for targeting cancer cells by using a fibronectin mimetic α5β1-selective RGD-containing peptide, named PR_b (60) (Figure 2). This group produced DPPC-based liposomal NPs covered by PEG and further decorated with PR_b peptide, and studied their targeting capacity in a CT26.WT mouse colon carcinoma experimental model. The quantities of PEG and peptide were fine-tuned in order to optimize the delivery of the nanovector. By increasing the quantity of conjugated peptide, an enhancement in binding of liposomes to cells was observed, whereas the opposite effect was found when the concentration of PEG was augmented. The cytotoxicity of 5-Fluorouracil carried by these PR_b targeted liposomes was found to be comparable to that of the free drug and better than that of the particles containing only the control GRGDSP sequence, confirming the importance of targeting α5β1 on this cancer model. Similar results were obtained in studies using HCT116 and RKO human colon cancer cells (60). This liposomal system has been further investigated for the delivery and cytotoxicity of DOX to MDA-MB 231 breast cancer cells (61). Confocal microscopy experiments showed that these targeted liposomes were internalized in breast cancer cells via an endocytic pathway, and transferred within the first minutes into early endosomes, and after prolonged times into late endosomes and lysosomes. Particularly at high concentrations, the therapeutic effect of encapsulated DOX in MDA-MB 231 cells was comparable to that of the free DOX.

In a recent approach, PR_b targeted polymersomes have also been explored for siRNA delivery (62). T47D breast cancer cells were studied to check the expression of *Orai3*. The downregulation of Orai3 levels results in cell apoptosis. The delivery of *Orai3* by PR_b-conjugated polymersomes decreased the viability of cancer cells but did not affect non-cancerous MCF10A breast cells. When compared to a commercial transfection agent (Lipofectamine RNAiMAX), the observed therapeutic effect of the polymersome formulation is still moderate. However, this method has not shown any systemic toxicity unlike other transfection reagents.

## Targeting the αvβ6 Integrin

The integrin subtype αvβ6 is expressed at low or undetectable levels in most adult epithelia, but may be upregulated during inflammation and wound healing (8). αvβ6 preferentially binds to TGF-β1 latency associated peptide (LAP) (63), but can also recognize the ECM proteins tenascin and fibronectin (64). In this regard, αvβ6 is biologically important for the activation of TGF-β1 and has been shown to control TGF-β activity or signaling in fibrosis and to play a crucial role in TGF-β-integrin crosstalk in carcinomas (65). Furthermore, αvβ6 was found to be significantly upregulated in tumor tissues (8) and in certain cancer types including colon (66), ovarian carcinoma (67), and in early stage of non-small cell lung cancer (NSCLC), which is associated with poor patient survival (68, 69). Other studies have shown that αvβ6 expression is correlated with the development of metastasis in gastric cancer and the enhanced survival and invasive potential of carcinoma cells (70, 71). This pathological relevance has turned αvβ6 into a promising target for tumor diagnostics and antitumor therapy.

To date, several linear and cyclic peptides as well as peptidomimetics have been developed to target specifically the αvβ6 integrin subtype (68, 70, 72–74). For instance, the high affinity αvβ6-specific 20-mer peptide H2009.1 (75) was conjugated as a tetramer to a poly-glutamic acid polymer carrying DOX, and was shown to specifically target αvβ6-expressing cells *in vitro* (76). In another work, the selectivity of this peptide toward αvβ6 was exploited to guide fluorescent quantum dots to lung adenocarcinoma cell line H2009 *in vitro* (68). Recently, this peptide has also been conjugated to a water soluble PTX conjugate resulting in selective cytotoxicity for the αvβ6-expressing NSCLC cell line (77). The conjugate was able to reduce the rate of tumor growth *in vivo*, however without an increased benefit over the use of free PTX. Furthermore, the same peptide was used to investigate the multimeric effect on functionalized liposomes (78). In this study, liposomes displaying tetramers of the H2009.1 peptide demonstrated higher drug delivery and toxicity toward αvβ6-expressing cells than liposomes displaying single copies of H2009.1, even if the total number of peptides bound to each liposome was identical. In another approach, H2009.1 was used to functionalize the surface of multifunctional micelles encapsulated with SPIO and DOX for MRI and drug-delivery applications, respectively (79). The functionalized micelles significantly increased cell targeting and uptake in αvβ6-expressing H2009 cells, as verified by MRI and confocal imaging.

A20FMDV2 (80, 81) is another αvβ6-selecitve 20-mer peptide (Figure 2) that can be used for targeted therapies. As an example, this peptide was radiolabeled on solid phase using 4-[^18^F]fluorobenzoic acid and the conjugate was selectively uptaken by αvβ6-positive tumors but not by αvβ6-negative tumors, as monitored in mice by PET (70). In a similar approach, A20FMDV2 was conjugated to 5-[^18^F]fluoro-1-pentyne via an azide-based 1,3-dipolar cycloaddition (click chemistry). However, no difference in tumor targeting *in vivo* was observed for such strategy compared to the previous labeling method (82). ^18^F-labeled derivatives of the same peptide were described to improve tumor uptake capacity in BxPC-3 (pancreatic cancer) xenograft-bearing mice over [^18^F]-FDG (83). Recently, A20FMDV2 was conjugated to an ^18^F-based tracer by copper-free, strain promoted click chemistry. However, the resulting derivative did not show a remarkable *in vivo* tumor uptake by mouse with mouse model DX3puroβ6-tumor (84). Furthermore, A20FMDV2 was conjugated to DTPA and labeled with ^111^In for SPECT imaging. In this study, the conjugate showed specific localization in αvβ6-tissues, and displayed increased uptake in an αvβ6-positive tumor and in a mouse xenograft model bearing breast tumors that express αvβ6 endogenously (85). Additionally, A20FMDV2 was incorporated into a recombinant adenovirus type 5 (Ad5) leading to increased cytotoxicity on a panel of αvβ6-positive human carcinoma cell lines *in vitro* and enhancement in tumor uptake and improved tumor transduction in an αvβ6-positive xenograft model *in vivo* over the Ad5 wild type (86).

In another approach pursued by the Gambhir research group, cystine knot peptides showing high affinity for αvβ6 but none for the related subtypes αvβ3, αvβ5, and α5β1 were developed and conjugated to ^64^Cu-DOTA for PET-based tumor imaging (87). Injection of these conjugates into mice bearing either αvβ6-positive BxPC-3 xenografts or αvβ6-negative tumors, and monitoring by PET imaging, showed αvβ6-selective targeting for the tumors expressing αvβ6. In a recent study (88), two cystine knot peptides were labeled with ^18^F-fluorobenzoate and their capacity to be uptaken by tumor cells assessed *in vivo*. PET imaging revealed for both peptides specific targeting of αvβ6-positive BxPC-3 xenografted tumors over αvβ6-negative HEK 293 tumors. These results illustrate the potential of the described strategies to be clinically used in PET imaging of αvβ6-over-expressing tumors.

## Concluding Remarks

A wide variety of carrier systems have been described to achieve tumor-specific therapeutic effects via integrin targeting. The principal success of this strategy is evidenced by two main observations – the dosage of drug has been usually reduced and an enhanced (and often selective) activity against tumors is achieved. The data obtained from independent studies using different carrier systems are promising and there is therefore hope to bring the targeted delivery methods into practice. However, a number of aspects related to the use of these drug-delivery systems in cancer therapy should be carefully considered.

In the first place, comparative studies between distinct carrier systems are missing. Such studies could provide useful insights on their relative advantages and disadvantages, and help in their further development and optimization. Detailed studies concerning the systemic toxicity and long-term side effects of the drug-delivery vectors in physiological systems are also essential. Another important aspect to optimize the concentration of drugs in cancer therapy would be to evaluate the efficiency of drug uptake with regard to the overall administered dose, but most studies have only rated the efficiency of the targeted systems in comparison to untargeted systems, without mentioning about the concentrations of the drug used. The investigation of the metabolic stability of these systems in gut and liver as well as their bioavailability profile would also be crucial to improve the efficacy of the therapy. Further optimization of such drug formulations could be directed toward new routes of administration, including, though certainly difficult, orally available conjugates.

It should be mentioned that most studies in this field rank the antitumor potency of the targeted systems based on the reduction in tumor volume and size, parameters that will however not entirely assure the success of the therapy. More satisfactory would be to carry out longer experiments to ensure the complete removal of tumors and arrest of resurrections. In this regard, recent findings have suggested that antiangiogenic therapeutics that aim at treating cancer primarily through reduction and control of tumor growth, may, in some cases, indirectly promote cancer invasiveness and metastasis (89, 90). This ultimately alarms development of targeted therapies which can inhibit multiple cellular functions and affecting not only cell survival *in situ* but also mechanisms involved in the promotion and progression of metastasis. Further investigations on this matter should include the study of targeted therapy on early stage and late stage tumors, and the effect (if any) of these strategies in the development of drug resistance mechanisms by some tumors. Additionally, treatment of cancer often necessitates a combination therapy (combination of different therapeutics or therapies). In this respect, it is demanding to study the usage of targeted approaches for delivering multiple drugs or therapies either by a single carrier system or multiple carrier systems. These studies are further pending in literature. Most of the studies on targeted gene delivery have used luciferase model system. Though it is a good analogous system for understanding gene delivery, proper experimental gene therapy studies aimed to treat cancers are to be extensively studied.

The choice of an optimal integrin ligand is another aspect of paramount importance in the design of integrin-based targeted therapies in cancer. This will depend on the differential pattern of integrin expression in cancer cell types and the biological activity and selectivity profiles of the targeting ligands. Many applications have used linear or cyclic RGD peptides to deliver drugs or nucleotides to tumors. Most of these peptides are active for αvβ3; however, it is often ignored that these ligands may target other integrin subtypes as well. This might not be relevant as long as simplified cellular or experimental animal models are investigated. However, it may raise safety concerns if clinical applications in humans are to be envisaged. E.g., the habitually used peptide – *c*(RGDfX), developed in our group long ago (25, 30), has about 1 nM affinity for αvβ3 and is certainly selective against αIIbβ3 (low affinity for the platelet receptor). Nonetheless, the compound also has affinity in the low nanomolar range for αvβ5 (7.6 nM) and α5β1 (15 nM) (73). Thus, the use of *c*(RGDfX) might not always provide enough selectivity to distinguish between distinct cell types. In this regard, our group has recently developed (91, 92) and functionalized (93, 94) peptidomimetics which can clearly discriminate between αvβ3 and α5β1. Application of such single integrin subtype selective ligands will enable a selective and controlled delivery of drugs to tumors, taking advantage of the distinct patterns of integrin expression found for each cancer type.

It is on the basis of these considerations that targeted therapy with integrin ligands be translated into clinical studies, and be demonstrated whether such strategy will result in a clear benefit for cancer patients.

## Conflict of Interest Statement

The authors declare that the research was conducted in the absence of any commercial or financial relationships that could be construed as a potential conflict of interest.

## Abbreviations

A549, human non-small cell lung carcinoma; ATCC, CCL-185 cells; ATCC, HTB-65 cells; bFGF, basic fibroblast growth factor; BMEC, brain microvascular endothelial cells; Cap-RGD, Ac-CCVVVTGRGDSPSSK-COOH; DCP-TEPA, dicetylphosphate-tetraethylenepentamine; DOPE, dioleoylphosphatidylethanolamine; DPPC, 1,2-dipalmitoyl-sn-glycero-3-phosphocholine; DSPC, distearoylphosphatidylcholine; DSPE, distearoylphosphatidylethanolamine; DTPA, diethylenetriamenepentaacetate; FDG, fluoro-2-deoxy-D-glucose; HEK, human embryonic kidney; HeLa, human cervical carcinoma cells; HPAE-co-PLA/DPPE, poly[(amine-ester)-co-(D,L-lactide)]/1,2-dipalmitoyl-sn-glycero-3-phosphoethanolamine copolymer; HPMA, *N*-(2-hydroxypropyl)methacrylamide; HAS, human serum albumin; HUVEC, human umbilical vein endothelial cells; IV, intravenous; Luc-pDNA, luciferase pDNA; Mal-PEG-PCL, maleimide-poly(ethylene glycol)-block-poly(ɛ-caprolactone); MDR, multi-drug resistance; MeWo, human malignant skin melanoma; MMAE, monomethyl-auristatin-E; MRI, magnetic resonance imaging; NSCLC, non-small cell lung cancer; PCL-PEEP, poly(ɛ-caprolactone)-block-poly-(ethyl ethylene phosphate); pCMVLuc, *Photinus pyralis* luciferase under control of the CMV enhancer/promoter; PEG, polyethylene glycol; PEI, polyethylenimine; PEO-b-PCL, poly(ethylene oxide)-block-poly(ɛ-caprolactone); PET, positron emission tomography; PGA, poly-glutamic acid; PLA, poly(lactic acid); PLG, poly-L-glutamic acid; PLGA, poly (D,L-lactide-co-glycolide); PLL, poly (L-lysine); PLys, polylysine; pORF-hTRAIL, plasmid expressing the tumor necrosis factor-related apoptosis-inducing ligand (TRAIL); P(PEGMEMA), poly[poly(ethylene glycol) methyl ether methacrylate]; sFlt-1, soluble fms-like tyrosinekinase-1 (pDNA encoding the soluble form of VEGF receptor-1); SPECT, single-photon emission computed tomography; TAT peptide, CGRKKRRQRRR; Tf, transferrin; TfR, transferrin receptor; TLT, transplantable liver tumors; VEGF, vascular endothelial growth factors.
